# Coronavirus Disease Contact Tracing Outcomes and Cost, Salt Lake County, Utah, USA, March–May 2020

**DOI:** 10.3201/eid2712.210505

**Published:** 2021-12

**Authors:** Victoria L. Fields, Ian T. Kracalik, Christina Carthel, Adriana Lopez, Amy Schwartz, Nathaniel M. Lewis, Mackenzie Bray, Carlene Claflin, Kilee Jorgensen, Ha Khong, Walter Richards, Ilene Risk, Maureen Smithee, Madison Clawson, Lee Cherie Booth, Tara Scribellito, Jason Lowry, Jessica Huynh, Linda Davis, Holly Birch, Tiffany Tran, Joseph Walker, Alicia Fry, Aron Hall, Jodee Baker, Eric Pevzner, Angela C. Dunn, Jacqueline E. Tate, Hannah L. Kirking, Tair Kiphibane, Cuc H. Tran

**Affiliations:** Centers for Disease Control and Prevention, Atlanta, Georgia, USA (V.L. Fields, I.T. Kracalik, A. Lopez, A. Schwartz, N.M, Lewis, T. Tran, J. Walker, A. Fry, A. Hall, E. Pevzner, J.E. Tate, H.L. Kirking, C.H. Tran);; Salt Lake County Health Department, Salt Lake City, Utah, USA (C. Carthel, M. Bray, C. Claflin, K. Jorgensen, H. Khong, W. Richards, I. Risk, M. Smithee, M. Clawson, L.C. Booth, T. Scribellito, J. Lowry, J. Huynh, L. Davis, H. Birch, A.C. Dunn, T. Kiphibane);; Utah Department of Health, Salt Lake City (J. Baker)

**Keywords:** COVID-19, contact tracing, respiratory infections, severe acute respiratory syndrome coronavirus 2, SARS-CoV-2, SARS, coronavirus disease, zoonoses, viruses, coronavirus

## Abstract

Outcomes and costs of coronavirus disease (COVID-19) contact tracing are limited. During March–May 2020, we constructed transmission chains from 184 index cases and 1,499 contacts in Salt Lake County, Utah, USA, to assess outcomes and estimate staff time and salaries. We estimated 1,102 staff hours and $29,234 spent investigating index cases and contacts. Among contacts, 374 (25%) had COVID-19; secondary case detection rate was ≈31% among first-generation contacts, ≈16% among second- and third-generation contacts, and ≈12% among fourth-, fifth-, and sixth-generation contacts. At initial interview, 51% (187/370) of contacts were COVID-19–positive; 35% (98/277) became positive during 14-day quarantine. Median time from symptom onset to investigation was 7 days for index cases and 4 days for first-generation contacts. Contact tracing reduced the number of cases between contact generations and time between symptom onset and investigation but required substantial resources. Our findings can help jurisdictions allocate resources for contact tracing.

By July 2021, >33 million cases of coronavirus disease (COVID-19), caused by severe acute respiratory syndrome coronavirus 2 (SARS-CoV-2), were documented in the United States, and most cases involved contact tracing by health departments (*1*). Preventing SARS-CoV-2 transmission through contact tracing requires rapid diagnosis, immediate isolation of cases, and rigorous tracking and precautionary isolation of close contacts (*2*–*4*). Because SARS-CoV-2 appears to be most transmissible before and immediately after symptom onset, clinical and transmission studies have shown that timely identification of cases and contacts is essential to preventing transmission (*5**–**7*). In addition, mathematical models have shown contact tracing, when implemented with other mitigation measures, can effectively reduce community spread of SARS-CoV-2 (*8*,*9*).

Evaluations of contact tracing for tuberculosis and HIV have found that contact tracing is an effective and sustainable approach to transmission reduction when disease prevalence is low but that contact tracing becomes less cost-effective as disease prevalence increases compared with other approaches, such as provider-initiated testing and intensified case finding (*10*,*11*). Programmatic data on outcomes and costs of contact tracing for COVID-19 are limited but essential for aiding public health agencies in designing or improving existing contact tracing programs (*12*). We aimed to quantify contact tracing efforts in Salt Lake County, Utah, USA, to examine how contact tracing affected case-finding, evaluate key contact tracing time intervals, and estimate the staff time and salary costs required to conduct investigations.

## Methods

We examined persons with laboratory-confirmed or probable COVID-19 cases and their close contacts retrospectively by using Salt Lake County Health Department (SLCoHD) surveillance data. We quantified the yield from each index case that generated a contact investigation and created transmission chains. We also examined 25 index cases and close contacts prospectively to estimate staff time and salary cost spent in contact tracing efforts.

### SLCoHD Contact Tracing Procedures and Testing Guidelines

During March 12–May 3, 2020, SLCoHD staff traced all reported case-patients with laboratory-confirmed SARS-CoV-2 infection and their close contacts. Close contacts of any confirmed or probable case-patients were traced until no further symptomatic or positive contacts could be identified. Early in the study period, state guidelines called for prioritizing testing symptomatic close contacts of confirmed COVID-19 case-patients. Later in the study period, testing was available to anyone with approval from their healthcare provider.

### Definitions

We defined a confirmed COVID-19 case as detection of SARS-CoV-2 RNA by real-time reverse transcription PCR (*13*). According to the Council of State and Territorial Epidemiologists definition, a probable case is one that meets clinical criteria and epidemiologic evidence with no confirmatory laboratory testing performed for COVID-19, meets presumptive laboratory evidence and either clinical criteria or epidemiologic evidence, or meets vital records criteria with no confirmatory laboratory testing performed for COVID-19 (*13*). We defined a probable case as a symptomatic close contact to a confirmed case-patient. We defined close contacts as anyone <6 feet of a confirmed case-patient or a symptomatic contact to a confirmed case-patient (i.e., a probable case) for >15 minutes, >2 days before the case-patient’s symptom onset and until the case-patient began strict isolation or until the contact’s last exposure to the case.

### Index Case Identification and Transmission Chains

SLCoHD staff conducted contact tracing investigations via telephone interview. Interviews included 5 components: providing isolation or quarantine guidance; monitoring contacts for 14 days after their last exposure to a case, with the option for daily phone calls or text messages; entering demographic data for contacts into the Utah National Electronic Disease Surveillance System (EpiTrax, https://epi.health.utah.gov/utah-national-electronic-disease-surveillance-system-ut-nedss) for linkage and tracking; community notifications, including notifying businesses, workplaces, event venues, churches, or persons who might have been exposed to confirmed cases; and providing resources, such as information on housing or financial support, SARS-CoV-2 testing locations, and where and when to seek medical care.

We grouped contacts into 3 main categories: confirmed cases, probable cases, and contacts under observation. We further divided the 3 categories into 8 subclassifications: confirmed cases comprised index, symptomatic positive, and asymptomatic positive cases; probable cases comprised untested but symptomatic persons; and contacts under observation comprised persons who were asymptomatic not tested, symptomatic negative, or asymptomatic negative, as well as unknown status cases (Appendix). Status of probable cases and contacts under observation could change during the quarantine period; for instance, a probable case could become a symptomatic positive case if the contact had a SARS-CoV-2–positive test result during the quarantine period.

### Data Source

We used EpiTrax surveillance data to retrospectively construct COVID-19 transmission chains for all confirmed index case-patients and contacts. We abstracted demographics, exposure history, SARS-CoV-2 test results, symptoms, and underlying conditions for confirmed or probable cases. We also abstracted investigation notes and applicable dates for last exposure to the confirmed or probable case, symptom onset, symptom resolution, initial health department contact, COVID-19 tests, monitoring period, hospital admission and discharge, and death. We also identified each contact’s relationship to their respective index case-patient, such as household or nonhousehold contact and generation of contact (first through sixth generation). 

We chose a priori to systematically select 10% of laboratory-confirmed cases diagnosed during March 12–May 3, 2020, in Salt Lake County. However, during that period, the number of cases identified in Salt Lake County grew. Our final sample represented 8% of the total 2,757 cases.

### Effort Time and Cost

We selected 25 index case-patients and prospectively documented the time spent interviewing them and their 144 contacts, from time of initial health department interaction with the index case-patient to the end of each contact’s 14-day monitoring period. Interviewers prospectively recorded time needed to complete all 5 investigation components for the selected index cas-patients. We grouped contacts into 1 of the 8 subclassifications and applied a β-PERT distribution to Monte Carlo simulation to estimate time and staff salary required to conduct contact tracing investigations for each of the 8 disease statuses (Appendix). We used the minimum, mean, and maximum time documented investigating each of the 8 disease subclassifications as parameters for the simulation (Appendix). We estimated salary cost by multiplying the median wage of all staff involved in contact tracing by the total number of hours spent on the contact tracing investigation (Appendix). Costs comprised time spent conducting all interviews (i.e., cost per index case and cost per contact, including those that were ultimately unreachable or out of jurisdiction) and for community notifications. We excluded nonstandardized costs, such as overhead, overtime, and time and costs for trainings.

### Data Management and Analysis

To quantify contact tracing efforts, we evaluated the number of contacts yielded and investigated from each index case. We did not reclassify symptomatic contacts to an index case-patient if their symptom onset date was earlier than their respective index case-patient, but we did include them in the analysis. We used R (R Foundation for Statistical Computing, https://www.r-project.org) and Stata (StataCorp LLC, https://www.stata.com) software for data management and descriptive analysis. We calculated 95% CIs for estimated time intervals between events, such as symptom onset, testing, and initial contact, and for estimated cost per type of case or contact investigation. This activity was reviewed by the Centers for Disease Control and Prevention and was conducted consistent with its policy and applicable federal laws (*14*–*19*).

## Results

### Index Case Identification and Contact Tracing

**Figure 1:**
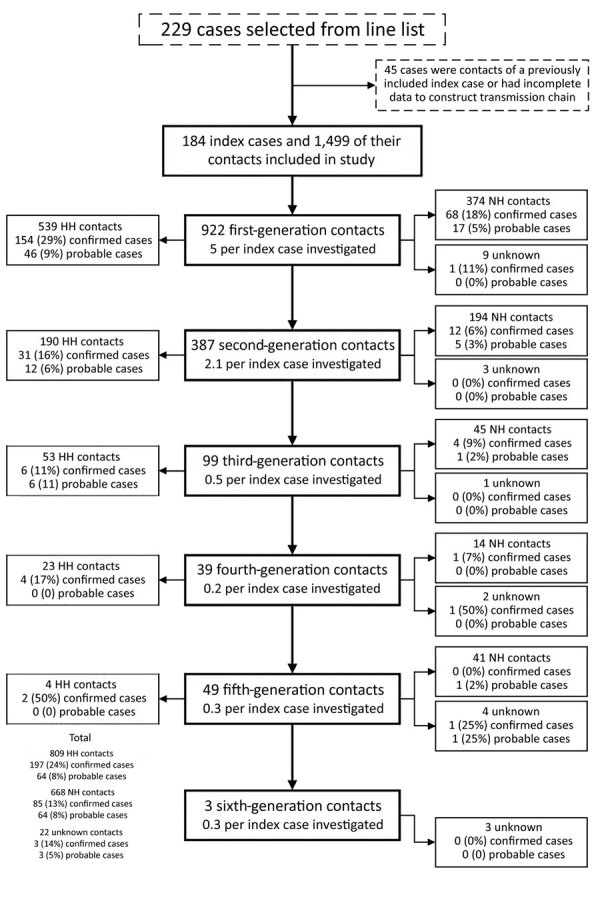
Flowchart of index case-patients and their contacts identified during coronavirus disease contact tracing, Salt Lake County, Utah, USA, March–May 2020. Confirmed cases comprised disease categories positive symptomatic, positive asymptomatic, and positive unknown symptoms. Probable cases comprised contacts in the not tested symptomatic disease category. Twenty-three HH contacts and 13 NH contacts were symptomatic on the same day or before the index case; contacts with an earlier symptom onset date were not reclassified. HH, household contacts; NH, nonhousehold contacts.

Of the 229 cases identified from the line list, 45 were excluded; 12 were excluded because the case-patient was a contact of a previously included index case and 33 because of incomplete data (Figure 1). Our final analysis included 184 index cases and 1,499 linked contacts. Among linked contacts, 922 were first-generation, 387 second-generation, 99 third-generation, 39 fourth-generation, 49 fifth-generation, and 3 sixth-generation contacts. Third-, fourth-, fifth-, and sixth-generation contacts were directly or indirectly linked to first-generation contacts of patients who tested positive, who had confirmed cases, or who had symptomatic but untested probable cases (Figure 1). Among 184 index case-patients, 153 (83%) did not have known contact with a laboratory-confirmed COVID-19 case-patient. Across all generations, we identified a median of 5 (range 0–97) contacts and a mean of 2.03 confirmed and probable secondary cases for each index case (Table). Of 1,499 contacts, 96 were unreachable; 89 were unreachable or did not have adequate information to trace, and 7 were out of jurisdiction and did not have final disease status. Of 1,499 contacts, 374 (25%) became confirmed or probable cases, of which 285 (19%) were confirmed and 89 (6%) were probable. The rate of secondary case detection was ≈31% among first-generation contacts; ≈16% among both second- and third-generation contacts; and ≈12% among fourth-, fifth-, and sixth-generation contacts.

**Table:** Number of contacts identified and COVID-19 status by generation among persons during COVID-19 contact tracing, Salt Lake County, Utah, USA, March–May 2020*

Generation	Final status†	No. (%)	No. contacts/no. index cases investigated‡	No. contacts traced to identify 1 case (mean)§
All, n = 1,499	Confirmed case	285 (19)	1.55	5.26 (4.01)
	Probable case	89 (6)	0.48	16.84 (4.01)
	Not a case	1,029 (69)	5.59	1.46 (NA)
	Unreachable or out of jurisdiction	96 (6)	0.52	15.61 (NA)
First, n = 922	Confirmed case	223 (24)	1.21	4.13 (3.22)
	Probable case	63 (7)	0.34	14.63 (3.22)
	Not a case	588 (64)	3.20	1.57 (NA)
	Unreachable or out of jurisdiction	48 (5)	0.26	19.21 (NA)
Second, n = 387	Confirmed case	43 (11)	0.23	9.00 (6.45)
	Probable case	17 (4)	0.09	22.76 (6.45)
	Not a case	304 (79)	1.65	1.27 (NA)
	Unreachable or out of jurisdiction	23 (6)	0.13	16.83 (NA)
Third, n = 99	Confirmed case	10 (10)	0.05	9.90 (5.82)
	Probable case	7 (7)	0.04	14.14 (5.82)
	Not a case	73 (74)	0.40	1.36 (NA)
	Unreachable or out of jurisdiction	9 (9)	0.05	11.00 (NA)
Fourth–sixth, n = 91	Confirmed case	9 (10)	0.05	10.11 (8.27)
	Probable case	2 (2)	0.01	45.50 (8.27)
	Not a case	64 (70)	0.35	1.42 (NA)
	Unreachable or out of jurisdiction	16 (18)	0.09	5.69 (NA)

*COVID-19, coronavirus disease; NA, not applicable. †Contacts were categorized as follows: confirmed cases comprised symptomatic-positive persons, asymptomatic-positive persons, and persons with unknown symptoms who tested positive; probable cases comprised symptomatic persons who were not tested; not a case comprised asymptomatic persons who were not tested, asymptomatic-negative persons, and symptomatic-negative persons. See Appendix for each generation breakdown by final status. ‡The number of contacts per index case investigated was calculated by dividing the number of contacts in each category by the 184 index cases. §The number of contacts traced to find a confirmed or probable case in each generation was calculated by dividing the total number of contacts (n = 1,499) by the number of cases in each category.

### Disease Status at Initiation and End of the Contact’s Monitoring Period

**Figure 2:**
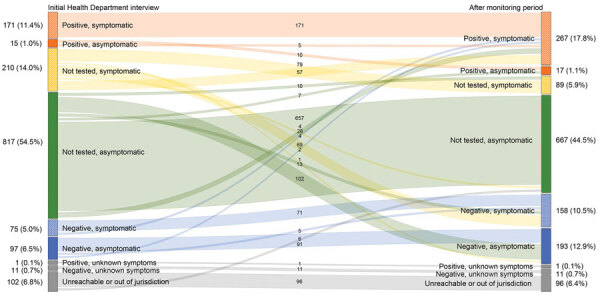
Coronavirus disease status at initial health department interaction and after 14-day monitoring period, Salt Lake County, Utah, USA, March–May 2020. Numbers in the center signify the change in status from initial interaction by health department after the monitoring period. Numbers on left and right represent total (%) of cases in each group. The median monitoring period was the time from initial health department interview to 14 days after the last exposure to the index case. Colors represent disease status classification category.

Among 1,499 contacts, 277/1,027 (27%) were tested during their monitoring period (Figure 2). Of the 277 tested contacts, 98 (35%) were SARS-CoV-2–positive after initial health department interaction. Among the 362 (24%) SARS-CoV-2–negative contacts, 183 (51%) had tested negative before their initial health department interview and 179 (49%) tested negative after the initial interview.

The proportion of household contacts who were symptomatic and positive increased from 11% at initial health department interaction to 18% after the monitoring period (Figure 2). When comparing the final disease status of contacts exposed within their household versus outside of their household, more contacts exposed within their households received testing (23% vs. 13%) (data not shown).

### Key COVID-19–Associated Dates

**Figure 3:**
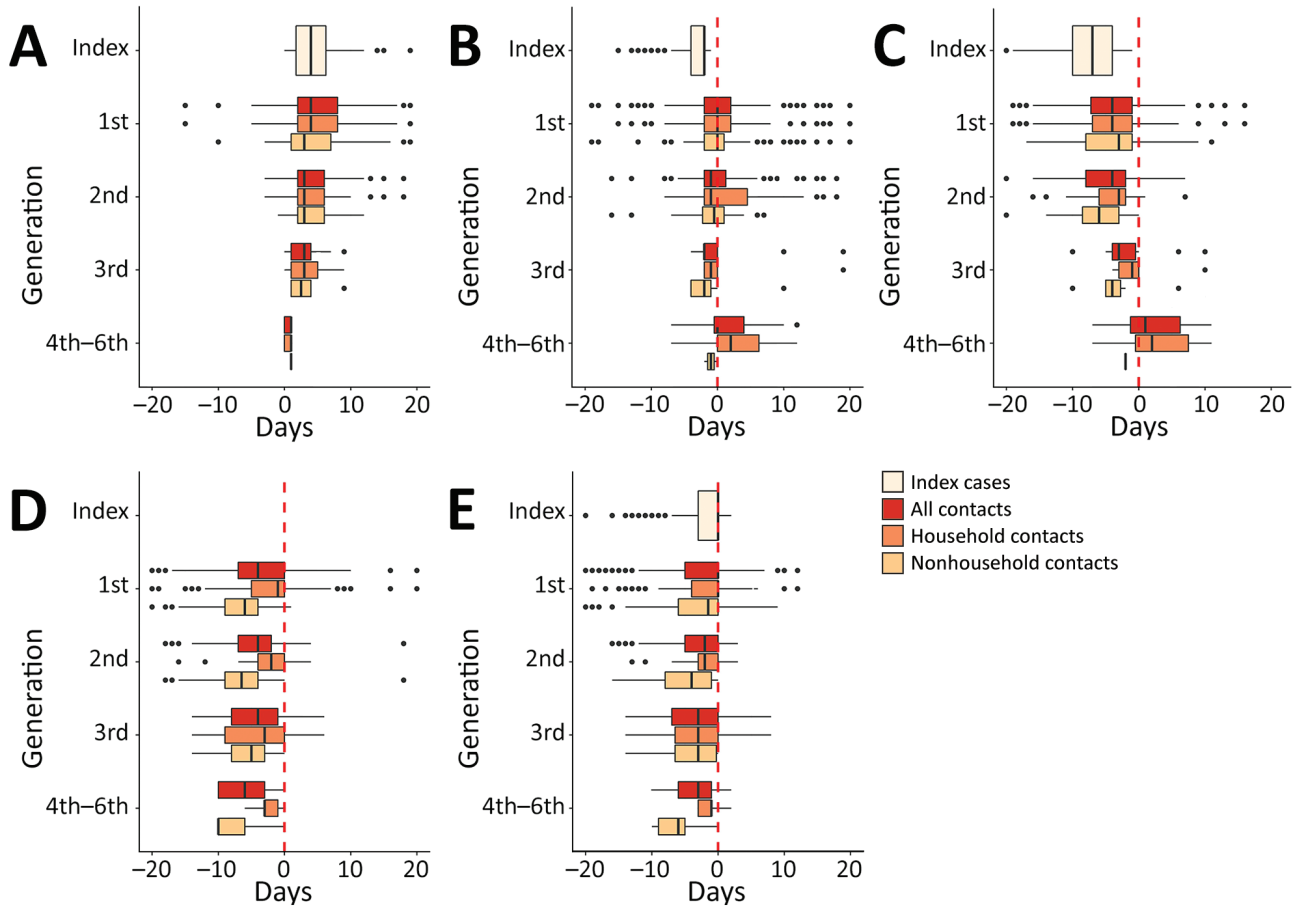
Box-and-whisker plots showing time from key coronavirus disease contact tracing–associated dates to other key dates, Salt Lake County, Utah, USA, March–May 2020. A) Days from symptom onset to PCR testing; B) days from PCR testing to initial interaction with Salt Lake County Health Department (SLCoHD); C) days from symptom onset to initial interaction with SLCoHD; D) days from last day of exposure to a confirmed or probable case to initial interaction with SLCoHD; E) days from monitoring start date to initial interaction with SLCoHD. The all contacts category includes contacts with an unknown relationship to a confirmed or probable case. Dotted red lines represent when the Salt Lake County Health Department had initial interactions with cases or contacts. Vertical lines within boxes indicate the median, left and right box edges indicate the interquartile range (IQR), and whiskers indicate the lower extreme and upper extreme quartiles; black dots indicate outliers. Negative values along the x-axis indicate that the second event happened before the first event.

The median time from symptom onset to initial health department interaction was 7 days (interquartile range [IQR] 4–10 days) for index cases compared with 4 days (IQR 1–7.25 days) for first-generation contacts (Figure 3; Appendix). The median time from laboratory PCR test collection to initial interview was 2 days (IQR 2–4 days) for index case-patients compared with 0 days (IQR 2–4 days) for first-generation contacts. Index case-patients generally started isolation on the day of the initial SLCoHD interview (median 0 days, IQR 0–3 days). First-generation contacts reported having quarantined themselves for a median of 0 days (IQR 0–5 days) before initial interview. First-generation contacts reported a date of last exposure as a median of 4 days (IQR 0–7 days) before the initial interview; household contacts reported a median of 1 day (IQR 0–5 days), and nonhousehold contacts reported a median of 6 days (IQR 4–9 days). The time between last exposure to isolation decreased for each subsequent generation (Appendix). Among 270 contacts who reported ongoing exposure, such as persons who could not or did not isolate, 96% were household contacts.

### Effort and Staffing Cost

**Figure 4:**
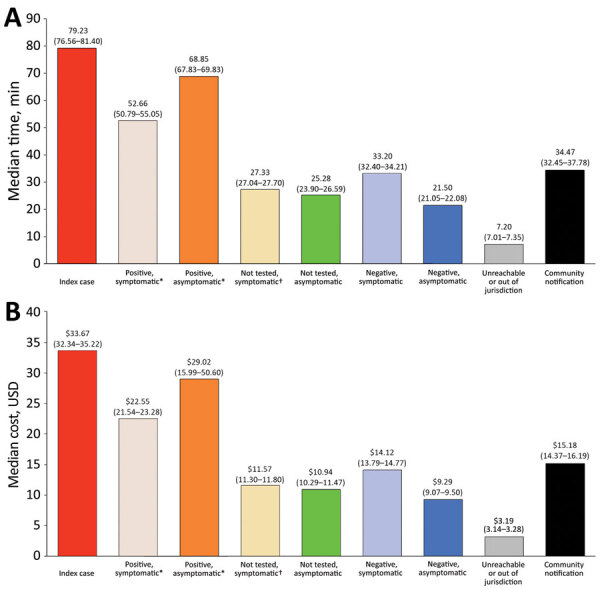
Estimated median time and cost spent educating, interviewing, and charting index cases and their contacts by final coronavirus disease status, Salt Lake County, Utah, USA, March–May 2020. Community notifications consisted of notifying businesses or persons that might have been exposed to the confirmed case such as in a workplace, at a wedding, or in a church. Asterisk (*) indicates case; dagger (†) indicates probable case. A) Median time in minutes and 95% CIs are reported above each bar. B) Median cost in USD and 95% CIs are reported above each bar.

We calculated time and salary cost (in USD) required to conduct contact tracing (Figure 4). Total time required to investigate 184 index cases and their 1,499 contacts was 1,102 staff hours at a total cost of $29,234 (Appendix). Median time and cost spent investigating an index case and all successive generations of contacts was 4.16 hours (95% CI 4.06–4.72 hours) at $107.22 (95% CI $92.60–$120.70). 

Time and costs varied depending on the status of the contact. For each index case, the median investigation time was 79.23 (95% CI 76.56–81.40) minutes and median cost was $33.67 (95% CI $32.34–$35.22). Negative asymptomatic cases required the least amount of staff time, 21.50 (95% CI 21.05–22.08) minutes costing a median of $9.29 (95% CI $9.07–$9.50). The total time spent on community notification for exposure to a confirmed case was 84.13 hours (Figure 4). Each notification took a median of 34.67 (95% CI 32.45–37.78) minutes, including 121 (66%) index case-patients who requested work excuse letters and 14 (7.6%) index case-patients who requested notifications to community locations, such as medical facilities, event venues, churches, and grocery stores. The average gross hourly wage for salaried epidemiologists, nurses, and office support staff involved in contact tracing efforts was $29.52 (range $23.61–$35.42) (Appendix Table 4).

## Discussion

Our analysis of contact tracing of 184 index cases and 1,499 close contacts in Salt Lake County, Utah, highlights the substantial cost and time needed for these investigations. In addition, we found that, for successive generations of contacts traced, fewer cases were identified, and the time between symptom onset and SARS-CoV-2 testing decreased. However, changing quarantine or social distancing guidance during the investigation period also might have resulted in fewer cases in later generations. These findings highlight the effectiveness of contact tracing to guide control measures and reduce onward transmission of SARS-COV-2. Other jurisdictions can use these findings to examine their contact tracing yields, effort, and key COVID-19–associated time intervals to help guide programmatic changes.

Contact tracing is resource intensive (*8*). Every index case investigated produced a transmission chain containing a median of 5 linked contacts. The median time to investigate these transmission chains was 4.16 (95% CI 4.06–4.72) hours at a cost of $107.22 (95% CI $92.60–$120.70). During the study period, 2,757 COVID-19 cases in Salt Lake County required investigation, which we estimate to have resulted in ≈$300,000 and ≈11,500 staff hours spent conducting these investigations. The time spent by contact tracers reflects resources needed to interview, educate, and enter data for cases and contacts and to write work excuse letters and conduct community notifications. The finding of lower yields in later generations highlights the need for further studies to examine the cost-benefit of tracing multiple generations of contacts (*20*).

We found that 6% of contacts were unreachable or out of jurisdiction, which is lower than the 17% unreachable contacts identified through a text messaging–based system in a previous study (*21*). However, consistent with another study (*22*), we found a high proportion (83%) of index case-patients that did not have known contact with a laboratory-confirmed COVID-19 case-patient. The prevalence of cases without an identified epidemiologic link raises concerns over unrecognized transmission (*23*), which suggests contact tracing efforts alone might not be sufficient to stop disease transmission.

Our contact tracing yields, laboratory confirmation of infection among 19% of contacts, were higher than those in South Korea (4%), and Shenzhen (15%) and Guangzhou (17%) in China (*2*,*7*,*24*). Consistent with findings from recent studies (*1*,*2*,*24*,*25*), we found household contacts were infected at a higher rate (32%) than nonhousehold contacts (16%). The finding of higher infection rates among household contacts reinforces the importance of evaluating prevention measures, such as using hotel services for contacts unable to separate themselves from other household members (*26*). Compared with index cases (n = 184), confirmed secondary cases (n = 285) identified through contact tracing generated about one fourth of the contacts and less than one fifth of the secondary cases. During the study period, testing capacity was limited, delaying health department notifications and initiation of contact tracing investigations, which might have increased yields because case-patients spent more time not knowing their infection status (*8*). In addition, because primarily symptomatic persons received testing, positive results might have resulted in higher rates and thus higher yields.

Modeling shows the probability of COVID-19 control decreases with long delays from symptom onset to case isolation, fewer cases ascertained by contact tracing, and increasing transmission before symptom onset (*8*). Thus, time intervals between symptom onset, laboratory testing, and initial health department interview provide insight into the efficiency of contact tracing investigations (*27*). One study found that contact tracing for COVID-19 reduced the time to test confirmation by 2.3 days and time to contact isolation by 1.9 days (*24*). Similarly, we observed a 3-day decrease in the time from symptom onset to initial health department interview starting with first-generation contacts and noted to be the same or further decreasing in most subsequent generations. The time interval from symptom onset to initial health department interview was longer than that from symptom onset to first positive test or from symptom onset to isolation initiation. This time interval decreased between the first-generation and sixth-generation contacts; later generation contacts might have had more opportunity to follow health department recommendations and for the health department to promptly recommend testing when indicated. Although the usefulness of contact tracing in the setting of sustained SARS-CoV-2 transmission has been questioned (*28*,*29*), consistent with other studies, our findings show that contact tracing reduced transmission; only one fourth of contacts traced and quarantined experienced COVID-19–like symptoms or tested SARS-CoV-2–positive.

New technologies, such as mobile telephone application–based symptom monitoring and electronic contact tracing platforms, might alleviate some of the burden needed to carry out investigations. In Utah, contacts could opt to receive daily phone calls or text message notifications. Text messaging might improve efficiency by decreasing time for contact follow-up, but it requires additional resources, a robust information technology infrastructure, and strong data protection safeguards (*21*). Smartphone technology is another powerful tool for contact tracing; a widely accepted smartphone application that does not have major privacy concerns, including the collection of personal data such as location, might prove useful (*30*). In addition, technology such as point-of-care SARS-CoV-2 testing, where results can be obtained within 48 hours, could reduce laboratory turnaround time. Rapid tests aid in quickly identifying index cases and contacts to implement isolation protocols (J. Joung et al., unpub. data, https://doi.org/10.1101/2020.05.04.20091231) and could improve contact tracing metrics. Online platforms that can identify how cases and contacts are linked, such as MicrobeTrace (https://microbetrace.cdc.gov/MicrobeTrace), also could aid in the management of investigations by reducing duplicative efforts, thereby improving efficiency.

The ongoing COVID-19 pandemic and emergence of the SARS-CoV-2 B.1.617.2 (Delta) variant have demonstrated the need for continuing layered prevention strategies, including contact tracing (*31*). Our findings can help local and state jurisdictions determine the cost, effort, and yields associated with implementing a comprehensive contact tracing program, factors that are crucial for guiding policy decisions. Our data, coupled with further cost studies, can help inform resource allocation, including staffing needs and roles, technology requirements, and strategies to evaluate cost-effectiveness. In addition, our findings can be used to develop mathematical models to determine the need to scale up contact tracing to focus on all cases and contacts or to scale down and focus only on first-, second-, and third-generation contacts, as well as to decide who to interview, such as high-risk contacts or household contacts.

Our study’s first limitation is that our approach might not be generalizable because Utah’s surveillance system enables linkage between cases and contacts, which might not be available in other jurisdictions; differences could also exist in contact participation across jurisdictions. Second, during March 2020, testing was available only for persons meeting initial COVID-19 symptom criteria (Appendix), which might have reduced case identification and the ability to test contacts. Third, interventions such as social distancing guidance and stay-at-home-orders introduced during March–May 2020 might have decreased transmission. Fourth, information was derived from interviews, which have a potential for recall bias, including naming all contacts (*32*). Fifth, costs of contact tracing are underestimated because we could not account for overtime benefits, such as time-and-a-half pay; overhead, such as staff health insurance and facility utility costs; staff training time; time spent providing services to the community, such as time to drop off masks; and other expenditures. Sixth, we could not track how many persons complied with recommendations to self-isolate or quarantine; the ability to determine whether cases and contacts complied with recommendations would aid in further quantifying contact tracing yield and effort. Finally, patients who do not seek care, potentially because of presymptomatic or asymptomatic infection, are a further challenge to preventing additional cases because SARS-CoV-2 shedding is highest early in illness (*8*). We found that 2% of asymptomatic contacts tested SARS-CoV-2–positive and 76% of asymptomatic contacts were not tested. Therefore, the attack rate might have been underestimated given the large proportion of asymptomatic contacts who did not get tested.

In conclusion, our analysis highlights the importance of contact tracing to reduce transmission of SARS-CoV-2. However, the effectiveness of contact tracing is contingent upon availability of substantial resources and rapid testing capacity. Persons should seek testing as soon as they experience COVID-19–like symptoms and begin isolation while results are pending. Because of early viral shedding, health department messaging should strongly direct contacts to obtain testing when possible, especially contacts with a higher risk for exposure, such as caregivers within households, populations in congregate settings, and contacts with underlying conditions; or for contacts who have an occupation requiring them to be in contact with other vulnerable persons, such as long-term care facility workers, daycare workers, and those who work with unvaccinated persons (*33*,*34*). Contact tracing metrics evaluated in this study can help other jurisdictions design, improve, and scale up contact tracing programs as needed for their specific epidemiologic contexts. Health departments should consider adjusting their approach to contact tracing as the situation evolves and adopting new technologies as these become available.

Appendix Additional information on coronavirus disease contact tracing outcomes and cost, Salt Lake County, Utah, United States, March–May 2020.
