# Supplementary material for: Coronavirus Disease Contact Tracing Outcomes and Cost, Salt Lake County, Utah, USA, March–May 2020
Source: Emerg Infect Dis. 2021 Dec;27(12):2999–3008. doi: 10.3201/eid2712.210505 (PMC8632199; doi:10.3201/eid2712.210505)
Supplement: Appendix — Additional information on coronavirus disease contact tracing outcomes and cost, Salt Lake County, Utah, United States, March–May 2020. [file 21-0505-Techapp-s1.pdf]

Article DOI: https://doi.org/10.3201/eid2712.210505

Coronavirus Disease Contact Tracing Outcomes and Cost, Salt Lake County, Utah, USA, March–May 2020

Appendix

Time and Cost

We grouped contacts into 3 main categories, confirmed cases, probable cases, and contacts under observation. We further divided the 3 categories into 8 subclassifications: confirmed cases comprised index case, symptomatic-positive, and asymptomatic-positive; probable cases comprised untested but symptomatic persons; and contacts under observation comprised asymptomatic not tested; symptomatic-negative; asymptomatic-negative; and unknown status.

To estimate the time and salary costs associated with investigating each classification, we used 10,000 iterations of a Monte Carlo simulation from a Beta-PERT distribution. The Beta-PERT distribution was developed in the context of Program Evaluation and Review Technique (PERT) and is parameterized by considering expert opinion on a minimum value, most likely value, and a maximum value. We prospectively documented the time staff spent interviewing 25 index cases and their contacts, from time of initial health department interaction with the index case, to the end of each contact's 14-day monitoring period. Interviewers prospectively recorded time needed to complete all 5 investigation components. We grouped contacts into 1 of 8 disease subclassifications. We took into consideration the minimum, most likely, and maximum values from the documented effort (Tables 1, 2). To construct 95% CI, we used a bias-corrected and accelerated bootstrapping procedure with 10,000 replicates by using R statistical computing version 3.5.2 (R Foundation for Statistical Computing, https://www.r-project.org) with boot and mc2d (*1*,*2*).

References

1. Canty AJ. Resampling methods in R: the boot package. The Newsletter of the R Project. 2002;2:3. https://www.researchgate.net/publication/284126128_Resampling_methods_in_R_The_boot_package

2. Pouillot R, Delignette-Muller M-L, Denis J-B, Pouillot MR. Package 'mc2d'. *hist.* 2017;1100:17.

Appendix Table 1. Contact tracing approach and isolation/quarantine guidance for 8 COVID-19 disease classifications, Salt Lake County, Utah, March–May 2020*

Category status of contact	Isolation/quarantine guidance	Contact tracing
Not tested, asymptomatic<br>Negative, asymptomatic	Contact is asked to complete 14 d of home quarantine from the date of last exposure to the case.<br>Contact is counseled to monitor for COVID-19 symptoms and to self-isolate if symptoms appear during the 14-d quarantine period.<br>Testing is highly recommended when symptoms appear.	Contact is followed passively for 14 d. Contacts are asked to notify investigator if COVID-19 symptoms appear. No contact tracing of this contact's contacts is conducted, unless the contact develops COVID-19 symptoms
Not tested symptomatic, probable case<br>Negative, symptomatic	Complete 14 d of home isolation from date of last exposure to case.	Contact tracing is required on this contact's contacts.<br>Contact is counseled to notify their close contact(s) of possible exposure and to monitor for COVID-19 signs and symptoms
Positive, symptomatic, confirmed case<br>Positive, asymptomatic, confirmed case<br>Positive, unknown symptoms, confirmed case	Strict isolation for 7 d with symptom improvement and fever-free for three days.	Contact tracing is required on this contact's contacts.
Negative, unknown symptoms	Complete 14 d of home isolation from date of last exposure to case.	Contact is counseled to notify their close contact(s) of possible exposure and to monitor for COVID-19 signs and symptoms.

*During the time of the evaluation, COVID-19 symptoms were fever, cough, or shortness of breath. COVID-19, coronavirus disease

Appendix Table 2. Number of COVID-19 contacts identified by generation and final status after 14-d monitoring period, Salt Lake County, Utah, March–May 2020*

Generation	Final status†	No. (%)	No. contacts/index case investigated, n = 184‡	No. contacts traced to identify 1 case (mean)§
First, n = 922	Symptomatic, positive confirmed case	207 (22)	1.13	4.45 (3.22)
	Asymptomatic, positive confirmed case	15 (2)	0.08	61.47 (3.22)
	Symptomatic, not tested probable case	63 (7)	0.34	14.63 (3.22)
	Symptomatic, negative	108 (12)	0.59	8.54 (NA)
	Asymptomatic, negative	122 (13)	0.66	7.56 (NA)
	Asymptomatic, not tested	356 (39)	1.93	2.59 (NA)
	Unknown symptoms, positive	1 (0)	0.01	922.00 (NA)
	Unknown symptoms, negative	2 (0)	0.01	461.00 (NA)
	Unreachable/out of jurisdiction	48 (5)	0.26	19.21 (NA)
Second, n = 387	Symptomatic, positive confirmed case	42 (11)	0.23	21.95 (6.45)
	Asymptomatic, positive confirmed case	1 (<1)	0.01	922.00 (6.45)
	Symptomatic, not tested probable case	17 (4)	0.09	54.24 (6.45)
	Symptomatic, negative	39 (10)	0.21	23.64 (NA)
	Asymptomatic, negative	48 (12)	0.26	18.82 (NA)
	Asymptomatic, not tested	212 (55)	1.15	4.35 (NA)
	Unknown symptoms, positive	0 (0)	0.00	NA
	Unknown symptoms, negative	5 (1)	0.03	184.40 (NA)
	Unreachable/out of jurisdiction	23 (6)	0.13	40.09 (NA)
Third, n = 99	Symptomatic, positive confirmed case	9 (9)	0.05	102.44 (5.82)
	Asymptomatic, positive confirmed case	1 (1)	0.01	922.00 (5.82)
	Symptomatic, not tested probable case	7 (7)	0.04	131.71 (5.82)
	Symptomatic, negative	9 (9)	0.05	102.44 (NA)
	Asymptomatic, negative	15 (15)	0.08	61.47 (NA)
	Asymptomatic, not tested	46 (46)	0.25	20.04 (NA)
	Unknown symptoms, positive	0 (0)	0.00	NA
	Unknown symptoms, negative	3 (3)	0.02	307.33 (NA)
	Unreachable/out of jurisdiction	9 (9)	0.05	102.44 (NA)
Fourth, n = 39	Symptomatic, positive confirmed case	6 (15)	0.03	153.67 (6.50)
	Asymptomatic, positive confirmed case	0 (0)	0.00	NA
	Symptomatic, not tested probable case	0 (0)	0.00	NA
	Symptomatic, negative	1 (3)	0.01	922.00 (NA)
	Asymptomatic, negative	7 (18)	0.04	131.71 (NA)
	Asymptomatic, not tested	19 (49)	0.10	48.53 (NA)
	Unknown symptoms, positive	0 (0)	0.00	NA
	Unknown symptoms, negative	1 (3)	0.01	922.00 (NA)
	Unreachable/out of jurisdiction	5 (13)	0.03	184.40 (NA)
Fifth, n = 49	Symptomatic, positive confirmed case	3 (6)	0.02	307.33 (9.80)
	Asymptomatic, positive confirmed case	0 (0)	0.00	NA
	Symptomatic, not tested probable case	2 (4)	0.01	461.00 (9.80)
	Symptomatic, negative	0 (0)	0.00	NA
	Asymptomatic, negative	0 (0)	0.00	NA
	Asymptomatic, not tested	34 (69)	0.18	27.12 (NA)
	Unknown symptoms, positive	0 (0)	0.00	NA
	Unknown symptoms, negative	0 (0)	0.00	NA
	Unreachable/out of jurisdiction	10 (20)	0.05	92.20 (NA)
Sixth, n = 3	Symptomatic, positive confirmed case	0 (0)	0.00	NA
	Asymptomatic, positive confirmed case	0 (0)	0.00	NA
	Symptomatic, not tested probable case	0 (0)	0.00	NA
	Symptomatic, negative	1 (33)	0.01	922.00 (NA)
	Asymptomatic, negative	1 (33)	0.01	922.00 (NA)
	Asymptomatic, not tested	0 (0)	0.00	NA
	Unknown symptoms, positive	0 (0)	0.00	NA
	Unknown symptoms, negative	0 (0)	0.00	NA
	Unreachable/out of jurisdiction	1 (33)	0.01	922.00 (NA)

*COVID-19, coronavirus disease; NA, not applicable.

†Confirmed cases are contacts in the following disease categories: Positive, Symptomatic; Positive, Asymptomatic; Positive, Unknown Symptoms. ‡ Probable cases are contacts in the following disease category: Not tested, Symptomatic. ¥ Not a case are contacts in the following disease categories: Not tested, Asymptomatic; Negative, Asymptomatic; Negative, Symptomatic.

‡The number of contacts per index case investigated was calculated by dividing the number contacts in each category by the 184 index cases.

§The number of contacts traced to find a case (confirmed or probable) in each generation was calculated by dividing the total number of contacts (n = 1,499) by the number of cases in each category.

Appendix Table 3. Time effort required for COVID-19 contact tracing for each disease classification used for β-PERT distribution Monte Carlo simulation, Salt Lake County, Utah, March–May 2020*

Final COVID-19 status	Average time, min	Minimum–maximum
Index case	63	32–210
Not tested, asymptomatic	16	2–97
Positive, asymptomatic	71	34–90
Negative, asymptomatic	20	10–40
Not tested, symptomatic	30	9–31
Positive, symptomatic	46.5	5–129
Negative, symptomatic	28	14.5–77
Notification	15	3–180
Unknown/unreachable	6	5–16

*Time effort was derived from a Monte Carlo simulation with a β-PERT to estimate the total time spent investigating the entire study population (n = 169) and median number of minutes spent investigating each of the 8 disease statuses. The β-PERT distribution was developed in the context of Program Evaluation and Review Technique (PERT). Parameters for the simulation were taken from the minimum, mean, and maximum time spent of each documented investigation of each disease status. COVID-19, coronavirus disease.

Appendix Table 4. Salary of health department staff involved in COVID-19 contact tracing, Salt Lake County, Utah, March–May 2020*

Staff classification	Average hourly wage	Range
Office staff	17.05	13.64–20.46
Office supervisors	23.24	18.59–27.89
STD investigators	26.98	21.58–32.37
Nurses	36.91	29.53–44.29
Nurse supervisors	43.41	34.73–52.09
Average	29.52	23.61–35.42

*Values are reported in US dollars. COVID-19, coronavirus disease; STD, sexually transmitted disease.

Appendix Table 5. Estimated cost of contact tracing for each disease classification, Salt Lake County, Utah, March–May 2020*

Final COVID-19 status	Median cost	95% CI	Total cost
Index case	33.67	32.34–35.22	6,541.53
Not tested, asymptomatic	10.94	10.29–11.47	8,184.48
Positive, asymptomatic	29.02	15.99–50.60	453.99
Negative, asymptomatic	9.29	9.07–9.50	1,927.83
Not tested, symptomatic	11.57	11.30–11.80	1,051.15
Positive, symptomatic	22.55	21.54–23.28	6,153.25
Negative, symptomatic	14.12	13.79–14.77	2,402.25
Notification	15.18	14.37–16.19	2,179.26
Unknown/unreachable	3.19	3.14–3.28	340.61

*Cost is calculated in US dollars. COVID-19, coronavirus disease.

Appendix Table 6. Estimated time in minutes of COVID-19 contact tracing for each disease classification, Salt Lake County, Utah, March–May 2020*

Final COVID-19 Status	Median time	95% CI	Total time
Index case	79.23	76.56–81.40	14,845.61
Not tested, asymptomatic	25.28	23.90–26.59	18,310.64
Positive, asymptomatic	68.85	67.83–69.83	1,148.39
Negative, asymptomatic	21.50	21.05–22.08	4,166.88
Not tested, symptomatic	27.33	27.04–27.70	2,404.63
Positive, symptomatic	52.66	50.79–55.05	14,168.9
Negative, symptomatic	33.20	32.40–34.21	5,230.33
Notification	34.47	32.45–37.78	5,048
Unknown/unreachable	7.20	7.20–7.35	786.89

*COVID-19, coronavirus disease.

Appendix Table 7. Days between key COVID-19 contact tracing–associated dates, Salt Lake County, Utah, March–May 2020*

Cases and contacts	Symptom onset and testing	Testing and initial contact	Symptom onset and LHD interview	Last date of exposure and initial contact	Monitoring start date and initial contact
Index case	4 (1.75–6.25)	−2 (−4– −2)	−7 (−10– −4)	NA	0 (−3–0)
First generation, all	4 (2–8)	0 (−2–2)	−4 (−7.25– −1)	−4 (−7–0)	0 (−5–0)
Household	4 (2–8)	0 (−2–1)	−4 (−7– −1)	−1 (−5–0)	0 (−4–0)
Nonhousehold	3 (1–7)	−1 (−2–1.25)	−3 (−8–1)	−6 (−9– −4)	−1.5 (−6–0)
Second generation, all	3 (2–6)	−1 (−2 –4.50)	−4 (−8– −2)	−4 (−7– −2)	−2 (−5–0)
Household	3 (2–6)	−1.5 (−2.25–1)	−3 (−6– −2)	−2 (−4–0)	−2 (−3–0)
Nonhousehold	3 (2–6)	−2 (−2–0)	−6 (−8.50– −3)	−6.5 (−9– −4)	−4 (−8– −1)
Third generation, all	3 (1–4)	−1 (−2–0)	−3 (−4– −0.50)	−6 (−8– −1)	−3 (−7–0)
Household	3(1–5)	−2 (−4– −1)	−1 (−3– 0)	−3 (−9– 0)	−3 (−6.5–0)
Nonhousehold	2.5 (1–4)	0 (−0.5–4)	−4 (−5–2.75)	−5 (−8– −3)	−3 (−6.5–0.25)
Fourth–sixth generation, all	0 (0–1)	2 (0–6.25)	1 (−1.25–6.25)	−6 (−10– −3)	−3 (−6– −1)
Household	1 (0–1)	2 (0–6.25)	2 (−0.50–7.50)	−3 (−3– −1)	−1 (−3– −1)
Nonhousehold	1 (1–1)	−1(−1.5–-0.5)	−2 (−2– −2)	−10 (−10– −6)	−6 (−9– −5)

*Results are median (interquartile range). COVID-19, coronavirus disease; LHD, local health department; NA, not applicable.

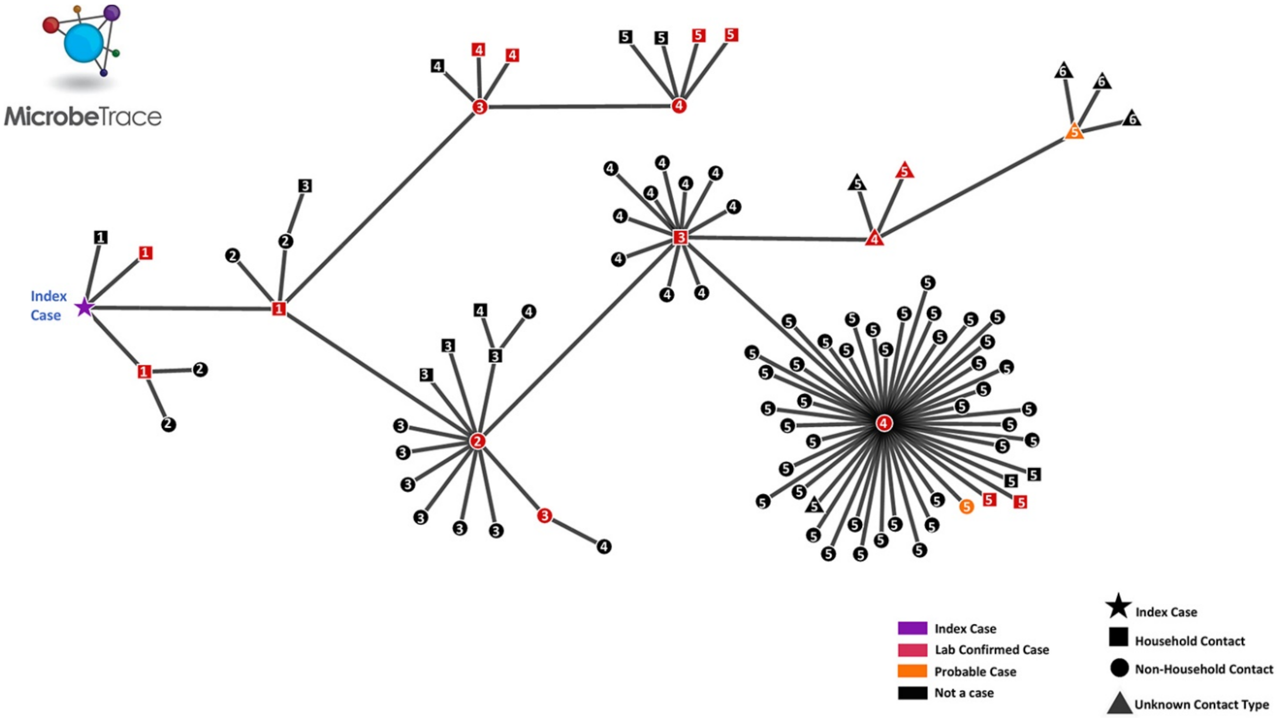

Appendix Figure 1. Transmission chain of 1 COVID-19 index case and first- through sixth-generation contacts identified during contact tracing, Salt Lake County, Utah, United States, March–May 2020. Nodes represent a contact; numbers within nodes denote the generation to which the contact belongs. Elongated nodes represent continued contact tracing and are not indicative of generation. During the evaluation period, Salt Lake County traced all contacts linked to laboratory-confirmed cases (red and purple nodes) and probable cases (symptomatic but not tested). Laboratory-confirmed and probable cases might not have contacts. Visualization created using MicrobeTrace (https://microbetrace.cdc.gov/MicrobeTrace).

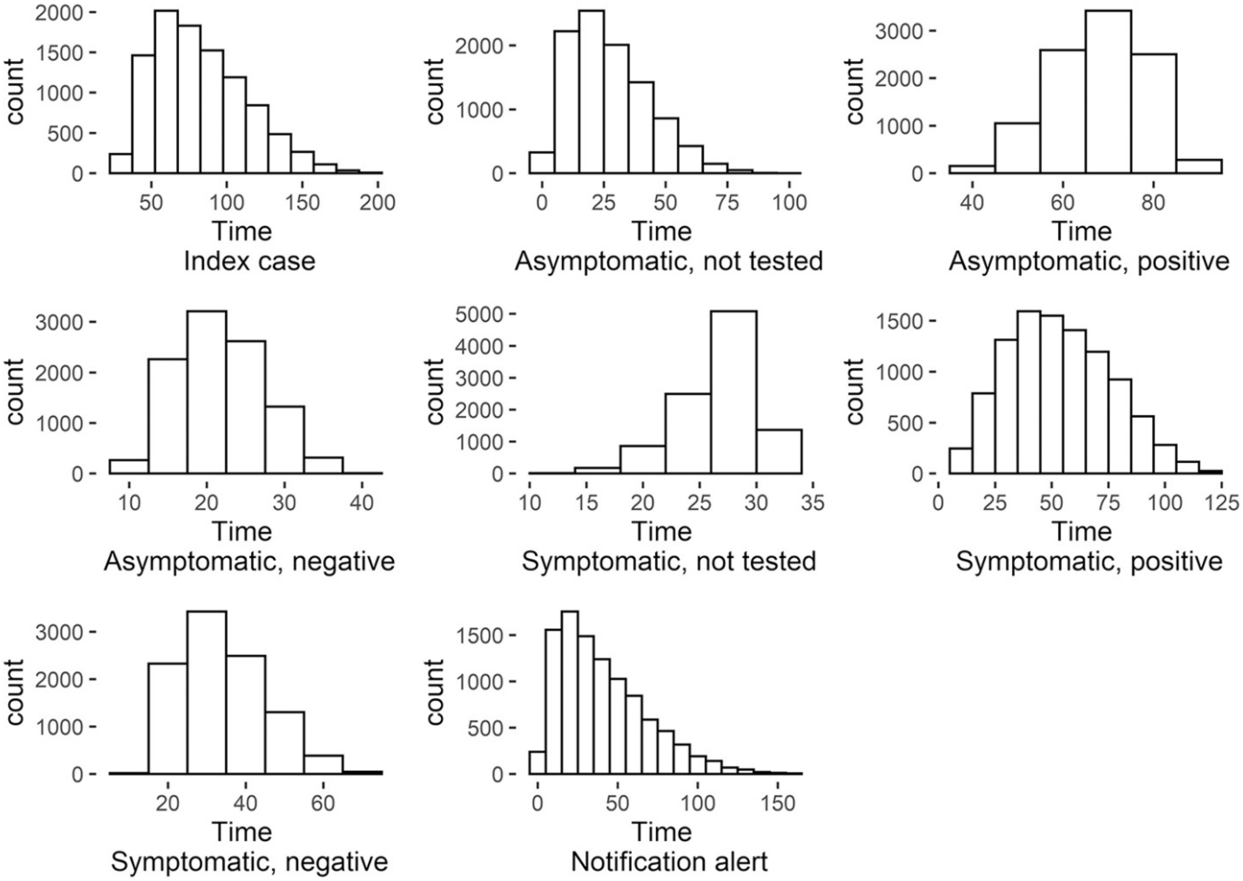

Appendix Figure 2. Estimated time needed to trace each of 8 disease classifications during COVID-19 contact tracing, Salt Lake County, Utah, United States, March–May 2020 obtained using 10,000 iterations of a Monte Carlo simulation from a Beta-PERT distribution. A) Index case; B) asymptomatic not tested cases; C) asymptomatic positive cases; D) asymptomatic negative cases; E) symptomatic not tested cases; F) symptomatic positive cases; G) symptomatic negative cases; and H) alert notifications for community venues visited by the index case. COVID-19, coronavirus disease.

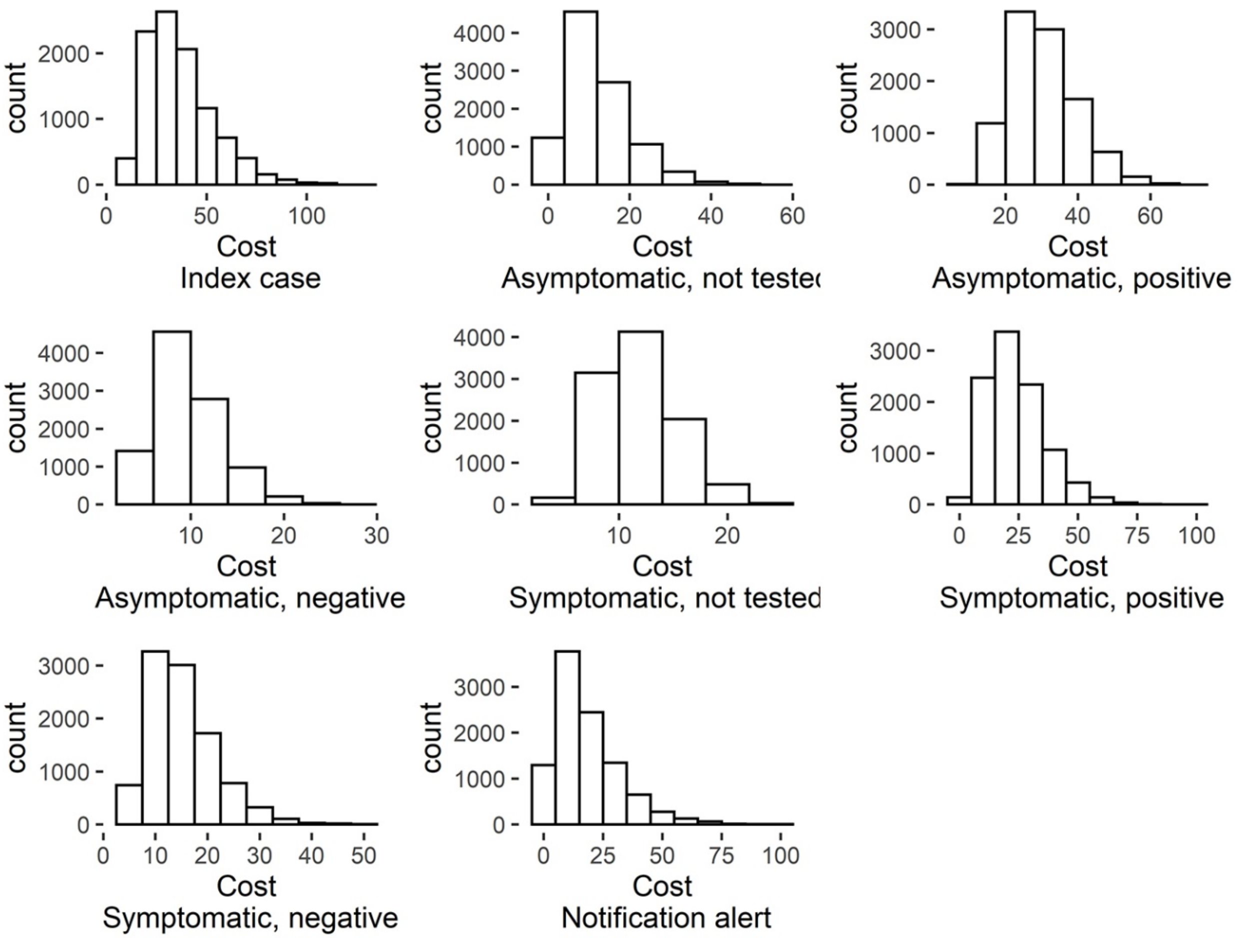

Appendix Figure 3. Estimated salary costs in US dollars for each of 8 disease classifications during COVID-19 contact tracing, Salt Lake County, Utah, United States, March–May 2020 obtained using 10,000 iterations of a Monte Carlo simulation from a Beta-PERT distribution. A) Index case; B) asymptomatic not tested cases; C) asymptomatic positive cases; D) asymptomatic negative cases; E) symptomatic not tested cases; F) symptomatic positive cases; G) symptomatic negative cases; and H) alert notifications for community venues visited by a COVID-19–positive case. COVID-19, coronavirus disease. during contact tracing for each disease classification.
